# Why children’s research assent matters: Exploring three dimensions of autonomy

**DOI:** 10.1177/09697330261424347

**Published:** 2026-03-02

**Authors:** Kajsa Norberg Wieslander, Tove Godskesen, Anna T. Höglund, Sara Frygner-Holm, Niklas Juth

**Affiliations:** 1 Department of Public Health and Caring Sciences, Centre for Research Ethics & Bioethics (CRB), 8097 Uppsala University, Uppsala, Sweden; 2 Faculty of Nursing and Health Sciences, 1786 Nord University, Bodø, Norway; 3 Department of Women’s and Children’s Health, 8097 Uppsala University, Uppsala, Sweden; 4 Department of Learning, Informatics, Management and Ethics (LIME), Stockholm Centre for Healthcare Ethics (CHE), Karolinska Institute, Stockholm, Sweden

**Keywords:** assent, children, clinical trials, healthcare professionals, informed consent, paediatric oncology

## Abstract

Children’s assent is an ethical and legal requirement for research that involves them. Nevertheless, studies suggest that children with cancer are not always involved in research decisions. This raises important ethical questions about how children’s participation in assent should be supported and on what ethical grounds. In this paper, we explore three dimensions of autonomy in research with children: autonomy as a right, as a value, and as relational. We argue that it is important not only to respect children’s autonomy but to also promote it as a value, by actively supporting children’s autonomy. Central to this approach is an understanding of how relationships and trust can both enable and constrain children’s autonomy. We suggest that children’s autonomy may be supported in practice through capacity-sensitive participation, child-adapted and iterative information provision. This requires ongoing dialogue that is responsive to children’s emotional, cognitive, and relational contexts, with particular attention to fears, decision-making challenges, and voluntariness. While concern for the well-being of children with cancer is essential, it should not be used as a justification to exclude them from the assent process. Respect for children as persons should guide recruitment to paediatric oncology research.

## Introduction

Paediatric oncology is described as a Learning Health System, where research and clinical care are tightly intertwined to develop better treatments and improve children’s quality of life. Families and professionals are generally committed to advancing paediatric cancer care, yet this integration creates unresolved value conflicts: how to safeguard children’s welfare while recruiting them into research, and how professionals should navigate their dual roles as clinicians and researchers.^1,2^

Recruitment to research is particularly sensitive because children are recognised as a vulnerable group requiring special protection.^3^ International guidelines state that their best interests must outweigh research aims,^4^ and paediatric research ethics has long stressed protection from harm, coercion, and undue risk.^5^ At the same time, the UN Convention on the Rights of the Child^6^ affirms children’s right to participate in decisions affecting them. This has increased the emphasis on seeking assent from children in addition to parental consent^4,7^ placing contemporary paediatric research ethics at the intersection of protection and respect for emerging autonomy.^8^ A central concern is how to address the vulnerability associated with serious illness, dependency, and care needs without allowing the duty of non-maleficence to override other fundamental ethical principles, including respect for autonomy and personhood.^9^ Related challenges include preserving trust between professionals and families without undermining informed consent,^2,10,11^ as well as paternalistic recruitment practices, such as the protective exclusion of children from assent discussions.^1,12^

In this article, we elaborate on the ethical basis of children’s assent to paediatric oncology research, mainly in terms of autonomy, with emphasis on school-aged children (7–14 years), who typically fall within the age range for assent. We do so by exploring three dimensions of autonomy in research with children: autonomy as a right, as a value, and as relational. Our aim is to develop an ethically well-grounded and empirically informed normative analysis of children’s autonomy in paediatric oncology research, and to indicate some practical implications for research practice that follow from this analysis. Unlike much of previous research studying children’s assent either empirically or theoretically, our approach integrates empirical insights with normative reasoning,^13^ making principles like autonomy more applicable in context.^14^ We also bring a care ethics perspective, emphasising relationships, trust, and responsibility,^15,16^ thereby framing autonomy as a right but also as a relational value that requires active support from parents and professionals. This goes beyond assent as a mere legal or procedural requirement. We focus on oncology because this is an area where we have identified new empirical findings of normative relevance and where our expertise lies. However, our arguments may also be relevant to paediatric research involving children more generally, or other paediatric patient groups.

## Children’s assent

Assent is the child’s affirmative and voluntary agreement to participate, based on age-appropriate information and a genuine understanding.^3^ It must be sought whenever the child is capable of giving it, while dissent must always be respected, and the absence of dissent should not be regarded as valid assent. International guidelines emphasise that assent should be based on meaningful participation, that is voluntary, informed, and tailored to the child’s evolving capacities, and supported by trained professionals in a safe environment.^3,4,17^ The World Health Organization (WHO) recommends involving children in assent from around age seven.^18^ Legal thresholds for obtaining consent (instead of assent) vary across Europe (14 to 18 years).^19^

Despite this consensus on the importance of assent, empirical studies report shortcomings. Many children with cancer are enrolled in research without their explicit assent,^20^ and up to one-third of school-aged children were not involved in the decision to participate in a randomised controlled trial, according to parents.^12^ Children have reported feeling excluded, unable to dissent, unaware of their research participation, and confusion due to complex language.^21,22^ Parents and professionals in paediatric oncology sometimes shield children from decision-making to protect them from distress, which may undermine their autonomy.^1,12^

## Autonomy

### Autonomy as a right

Respect for autonomy, a cornerstone principle of research ethics, is traditionally framed as the right to self-governance, freedom from external control and interference. In research, respect for autonomy is closely linked to informed consent, which is not only a legal requirement but a moral one grounded in respect for persons, including respect for autonomy. Consent procedures are an essential means of expressing such respect and play a crucial role in preventing the exploitation or manipulation of vulnerable research participants.^23^ Autonomy is often interpreted primarily as a negative right – that is, a right to freedom from undue influence by others, it involves respecting the individual’s right to make free and informed choices about research participation.^14^ The question that arises, however, is whether a child has the capability to execute such a right.

Research shows that children are often capable of engaging in research decisions when adequately supported. For instance, children as young as 12 often demonstrate cognitive capacities for informed consent comparable to those of adults.^24^ Even younger children aged 5–7 years can understand key research concepts and express preferences about research.^25^ Crane et al. (2019) found that children over 10 without cognitive impairment were considered capable of deciding on participation in early-phase oncology trials, when supported by their parents.^11^

Regardless of their level of decision-making capacity, children should be treated with respect for their wishes and perspectives. This aligns with autonomy as a right, but also with the principle of integrity, which protects personal privacy and the right to have one’s preferences respected, especially in matters involving the body and self. Integrity is closely tied to human dignity and is particularly relevant for children.^26^ Medical research can impact children’s well-being and impose burdens such as procedures and hospital visits.^11^ Following Grill (2020), children’s preferences should be respected wherever reasonably possible, even if they cannot fully grasp all aspects of the decision.^27^ This includes honouring children’s dissent, or ‘veto-right’, even when parents have given consent.^4^ Tait and Geisser (2017) contend that a basic (rather than complete) understanding of how research participation may affect oneself is sufficient for child assent procedures to be meaningful.^28^

Respecting children’s autonomy as a right does not mean shifting full decision-making responsibility onto them. Rather, it highlights the ethical obligation to engage with them in ways that respect their actual capacities. Many children prefer collaborative decision-making with parents and professionals over independent choice.^21^ Children’s willingness to actively and meaningfully participate varies by age, personality, health, and context, and researchers and research nurses should actively explore each child’s preferences without imposing on or excluding them.^29^

Autonomy also entails the right not to participate in decision-making.^14^ Children may choose to defer or opt out of research decisions, and this should be the child’s voluntary choice – one made free from pressure or lack of opportunity. While children have the right to delegate decisions, parents have a duty to safeguard their children’s interests, which necessitates that parents are reasonably well-informed. Therefore, researchers and research nurses must ensure that parents receive assessable and adequately accessible information adapted to their level of understanding, including considerations such as their educational background.^30^ In conclusion, respecting children’s autonomy as a right requires considering their actual capacities, wishes, and perspectives, including their right to not be involved in decisions – while ensuring that parents are adequately informed to decide.

### Autonomy as a value

In the previous section, we have discussed autonomy as a right that children are entitled to.^14^ However, autonomy can also be perceived as a value that should be actively promoted; this introduces the notion of positive rights, for example, the right to receive support in the development of one’s decision-making capacities.^14,31^ In paediatric research, this distinction carries significant implications: researchers and research nurses should not only respect children’s decisions but also actively support their autonomy. This entails actively fostering conditions that enable meaningful participation and removing barriers that hinder it. This is especially important for children diagnosed with cancer as they rely much on adults for support.^30^

As a child’s autonomy develops, their participation in decision-making concerning research should reflect their growing capacity and authority.^4^ While many feel overwhelmed early in treatment, they often become more capable of engaging in complex decisions over time. Yet, children with cancer are sometimes excluded even in the late stages of illness, when participation is most critical, for example, in choices about aggressive or experimental therapies.^32^ A common view holds that low-risk decisions require less capacity, while high-risk decisions demand greater maturity.^14^ Although this seems reasonable, it risks reinforcing paternalism and denying children their right to participation: children may be excluded from important decisions altogether not because they lack competence, but because professionals deem their capacity as insufficient. We argue for the contrary, children facing relapse or poor prognosis – where stakes are highest – should be granted greater, not lesser, participation and authority in decision-making. This view is supported by findings that parents often want their child’s preferences to carry decisive weight in such circumstances.^11^ Promoting autonomy as a value therefore requires actively eliciting and prioritising children’s voices, not least during critical moments. Balancing a child’s need for protection with respect for their autonomy can however be challenging in certain situations – a point that we elaborate on below.

### Conflicts between respecting and promoting autonomy

As has been noted by others, tensions may arise between respecting a child’s present autonomy and safeguarding their future autonomy.^33^ This might occur when a child opts out of a study aimed at preserving cognitive function and that decision would compromise their future decision-making capacity. Such situations place parents and researchers in a challenging position: they must decide whether to respect the child’s current preferences or act paternalistically in service of their future autonomy by protecting their cognitive capacity. It may be ethically justifiable to override a child’s refusal – but such decisions require careful ethical justification. The legitimacy of paternalistic intervention hinges on the child’s decision-making capacity. The more competent and informed the child is, the stronger the reasons to respect their choices – even if those choices involve risks to their future autonomy.^27^ Capacity assessments may be essential in these situations, and active measures may be especially crucial to support the child’s autonomy as a value, by making them better equipped to engage in impactful and critical decisions. What this support can entail in practice, is elaborated below.

### Supporting the value of children’s autonomy in practice

As mentioned, structured capacity assessments may be needed to ensure that the child’s participation in decisions reflect the child’s actual capacity. Children’s capacities should always be assessed in context, and focus on the specific demands of the research decision in mind, rather than the child’s general maturity.^14^ Capacity assessments can be supported by validated tools, like the MacArthur Competence Assessment Tool for Clinical Research (MacCAT-CR).^24^ Researchers and research nurses should start from the presumption that each child has some capacity to participate and justify exclusion carefully. Capacity assessments should not serve as gateways, but instead inform the degree and tailoring of children’s participation. Only in exceptional cases – such as when dealing with infants who clearly lack the capacity to make informed choices – should parents be sole decision-makers, guided by the child’s best interests.^34^

The fact that a child’s illness can lead to increased dependence and isolation makes recognition and empowerment vital in terms of supporting children’s autonomy. As suggested by Rogers, Mackenzie, and Dodds (2012), autonomy-supportive measures are particularly important to protect and empower individuals who are inherently or situationally vulnerable.^35^ While physicians often defer to parents in decision-making, research highlights the importance of engaging children directly to build confidence and a positive sense of responsibility.^36–38^ Creating a safe, inclusive environment is important, and individual conversations may be necessary when parents undermine children’s participation.^11^

To support children’s capacity to decide, emotional and cognitive challenges need to be addressed. For example, cognitive difficulties due to the illness may present.^14^ Further, emotional distress, fear, and cognitive overload are common at diagnosis.^22,39^ Specific fears related to research participation, such as concerns about side effects or receiving suboptimal care, may also arise.^12^ Children and parents may hide or suppress fear to protect one another, and researchers may not always recognise this. Emotional defence mechanisms – such as relying on general reassurances while neglecting critical information – can prevent genuine understanding.^22,39^ In addition, misconceptions about the therapeutic benefits of research participation are common. For example, children and parents often believe that participation will enhance care or safety.^40^

Additional strategies may enhance autonomy when informing children about research participation, including step-wise information provision, the repetition of key messages, emphasis on voluntariness and the clarification of expectations regarding benefits and limitations. Researchers should avoid overstating the benefits of paediatric oncology research or using emotionally charged appeals, as these may unduly influence a child’s willingness to participate.^22,40,41^ In high-risk cancer situations, clear and honest communication paired with empathetic support is especially important.^11^ Those recruiting children with cancer should approach families with empathy, flexibility, and responsiveness to each child’s emotional and cognitive state.^1,2^

Other well-known obstacles to autonomy include language barriers affecting research information. These barriers may result from the use of complex medical language and references to legislative requirements, and can be exacerbated for non-native families.^2^ To enhance children’s understanding and overcome language barriers, age-informed tools such as those developed by Lepola et al. (2022) can aid in tailoring assent for different age groups.^19^

While child-adapted information is crucial, it is insufficient to fully support a child’s autonomy. Researchers also have a responsibility to ensure that children genuinely understand the information provided. This requires a dynamic, dialogue-based approach.^2^ Practically, it may include strategies like the teach-back method, whereby children are encouraged to repeat the information in their own words, allowing the researcher to assess the child’s understanding and correct any misunderstandings. Promoting children’s autonomy through dialogue is not only important during initial assent. Sustained communication and support may be needed throughout the research process to identify shifts in the child’s willingness to participate and new concerns that may arise as the child’s understanding evolves. A research environment that is sensitive to children’s evolving informational needs and concerns is essential, especially when research participation stretches over time.^10,42^

Understanding autonomy as a value to be promoted and supporting it in practice, shifts the focus from assent as a one-time event to a dynamic continuous process, with emphasis on not only who makes the decision but also how it is made.^32^ This necessitates reflexivity regarding the quality and meaningfulness of children’s participation and the support offered to them in terms of communication, emotional responsiveness, and attentiveness to their evolving needs and capacities. This view aligns with United Nations Convention on the Rights of the Child, which emphasises children’s full personhood and calls for respect for their universal rights beyond minimal legal assent requirements, which may vary significantly across jurisdictions.^19^

### Children’s autonomy as relational

The concept of relational autonomy provides a valuable lens for understanding paediatric assent, complementing and extending traditional notions of individual autonomy. While traditional conceptions of autonomy emphasise independence and self-governance, relational autonomy underscores how children’s decision-making is shaped by relationships, emotional support, and the social context.^43–45^ This is particularly relevant in paediatric oncology, where decisions unfold within a triadic constellation of child, parents, and clinicians and researchers. These relationships – marked by emotional bonds, trust, and asymmetries in knowledge – significantly shape how children experience and exercise their self-determination.^46^ In other words, a child’s autonomy cannot be understood solely in terms of cognitive capacity – it is also relational and dependent on social dynamics, including the attitudes, communication, and behaviours of adults.^33^

Wilkinson and Levy’s (2024) concept of ‘scaffolding’ offers a valuable way to operationalise relational autonomy within paediatric assent. Although not initially designed for children, scaffolding highlights the role of support structures and strategies that allow children to take an active and meaningful part in decision-making. Trusted relationships can function as supports or ‘scaffolds’ for children, meaning that through dialogue, explanation, and emotional attunement, adults can help children understand complex information, articulate values, and make decisions that reflect their authentic preferences. Importantly, in children, these capabilities often emerge precisely through dialogue with trusted adults.^47^ Empirical research supports this view; researchers, and especially nurses who listen actively, validate children’s perspectives, and build trusting relationships enhance children’s sense of safety, reduce knowledge asymmetry, and help children with cancer feel more in control and supported in their decisions.^37,48^

However, children’s relationships can also constrain autonomy, rather than support it. Children may feel pressured to participate in research due to the expectations of parents or researchers, and adults may impose values that are incongruent with the children’s own.^47^ While some studies report that children with cancer may feel obligated to partake in research, others state that they do not necessarily feel pressured, despite being recruited by professionals involved in their care.^37^

Some decisions – for example those involving sensitive personal issues or palliative care – are what Wilkinson and Levy (2024) term ‘autonomy-critical’, meaning they should ideally reflect the child’s authentic will, free from undue external influence.^47^ In such decisions, it is especially crucial to identify negative relational autonomy-constraining influences, for example from family dynamics. Explicitly acknowledging these risks during assent conversations is crucial. Professionals and researchers should explore how the child perceives their relationships, that is, as autonomy-supporting or constraining. For instance, if the child feels involved and mandated to voice their concerns, or instead feel marginalised in the process, and pressured or persuaded to participate. It is also important to consider how broader cultural, legal, and social norms, practices, and beliefs encourage or restrict children’s participation and autonomy in assent.^19^

Adopting a relational perspective on autonomy includes the recognition that research decisions may impact parent–child relationships in both the short and long term. Communication in paediatric oncology serves multiple purposes: it not only conveys information but also builds trust, offers emotional support, and promotes well-being.^49^ Children’s participation is not merely a matter of making choices but also affects how they view themselves and their relationships. Moreover, the families are those who live with the consequences of these decisions, and both parents and children may regret participating in research.^50^ Shared decision-making – where parents and children are engaged as collaborative partners – is more likely to yield decisions that both parents and children find acceptable.^42^ Viewing children’s autonomy as relational involves the recognition that a child’s autonomy depends not only on cognitive capacity but also on the relational and emotional aspects of decisions as they unfold. In particular, relationships with trusted adults can serve both as autonomy-scaffolds and as autonomy-constraints for children in assent. Given the prominent role that trust plays in children’s relationships and the context of assent, it will be devoted a separate section below.

## Trust: A prerequisite and ethical challenge

So far, we have argued, that autonomy in children’s assent is best understood as a value and as a relational activity, rather than as merely a right. A relevant ethical aspect in this activity is trust. For example, while the dual roles of healthcare professionals/researchers may enhance trust and promote children’s active engagement,^10^ they may also introduce ethical problems related to trust. Families rely on medical expertise to help them act in their child’s best interests, often assuming that healthcare professionals will not propose harmful research. This trust can simplify decisions and reduce distress for parents, particularly in complex scenarios like randomisation.^30,37,42^ However, it may also lead to parents giving their consent despite uncertainty or limited information.^2^ Trust in the medical team further influences how openly parents express concerns and whether children feel empowered and confident participating in assent.^11^ Feeling excluded and overlooked in decision-making about research can damage children’s trust.^29^ It is evident that trust enables ethical research conduct and well-being, yet it also carries ethical risks – highlighting the need for transparent communication regarding risks and uncertainty as well as safeguards to protect children’s interests.^40,42^ Awareness of both the positive and negative aspects of trust in paediatric oncology research assent is important.

## Respect for personhood and risk of instrumentalisation

A key ethical concern in paediatric research is the risk of instrumentalising children who cannot provide informed consent, particularly as they often receive no direct benefit from experimental studies. This raises significant concerns about children being treated as mere means, rather than ends in themselves.^51^ If children are not meaningfully involved in assent, with sincere consideration of their evolving autonomy, personal goals, and experiences, they risk being instrumentalised, overshadowing their recognition as persons in favour of research aims.

Assent has been proposed as a means of acknowledging children’s moral status in the absence of legal consent.^52^ However, it only serves this purpose if it genuinely engages the child as a rights-bearing individual. When assent becomes a mere procedural formality, something that is to be ‘obtained’ but that disregards children as persons, it risks perpetuating instrumentalisation rather than preventing it. To counter this, assent procedures should encourage active and meaningful participation and recognise children as autonomous individuals within their developmental capacities. Children should be viewed not as incomplete adults, but as persons with evolving abilities, values, and aspirations. While children’s participation in research may support their moral development – for example, by fostering altruism – this alone does not safeguard against instrumentalisation.^51^

Despite the legal recognition of children’s rights in many countries, children’s status as independent rights-holders remains insufficiently acknowledged in practice.^17^ Therefore, it is especially important that paediatric oncology research is framed as a reciprocal, voluntary partnership in which children are morally recognised and seen as active contributors. This aligns with the values put forth by the European Society for Paediatric Oncology.^53^

## Paternalism and well-being concerns

We have argued that children should be supported to engage in the assent process concerning research in paediatric oncology. One reason why this is not always done, is the will to protect children and safeguard their well-being by excluding them from difficult decisions; in other words, through acts of paternalism. Paternalism is defined as the act of overriding an individual’s autonomy for the purpose of preserving their perceived well-being or preventing harm.^14^ Examples of paternalism include children being excluded from research discussions to protect them from distress and burden,^12,54^ and research decisions being made on behalf of emotionally fragile families.^1^ Refraining from obtaining a child’s assent on the basis of vulnerability by definition constitutes paternalism when decisions are made on the child’s behalf, either by (1) excluding the child from decision-making altogether or (2) overriding the child’s expressed preferences in the name of their best interests.^14^ While often motivated by care and concern, these may be acts of misdirected protection that undermine children’s active and meaningful participation. Justification of unwarranted paternalism is a common critique of the ethics of care, because of the strong emphasis on care.^15^ However, care ethicists tend to be critical towards paternalism themselves.^16,55^ Protective exclusion is predicated on the assumption that distress makes individuals incapable of deciding. Indeed, poor health status and feeling overwhelmed – particularly shortly after a diagnosis – may compromise a child’s and parent’s capacity.^1,12,54^ However, rather than excluding, we argue for ‘scaffolding’ measures that actively support children and parents in making informed choices, even in emotionally burdensome situations.^47^

While paternalism against children may in many circumstances be morally warranted, there are ethically significant reasons to argue for supporting the value of autonomy in its relational form rather than excluding children from decision-making, particularly in research. The conventional distinction between care and research^3^ holds that care prioritises the child’s best interest, while research entails uncertainty and primarily seeks to benefit future patients. Since many studies are not designed to improve the child’s own health, participation may yield no direct benefits and may involve substantial risks, side effects, or burdens. Therefore, the ethical justification for enrolling a child with cancer in research relies heavily on voluntariness, respect for the child’s personhood and their emerging autonomy – making the child’s active and meaningful participation in assent especially significant. In exceptional circumstances, research may appear to offer a unique potential benefit for the child. However, because the benefits of research are always uncertain, the ethical justification for paternalistically overriding a child’s wishes in their best interest remain highly contested.^3^ A practical implication is therefore that to truly respect children as persons and their developing autonomy, their dissent should always be respected in research. Children may prefer to delegate decision-making to their parents or trusted adults and should always have that choice respected. Such delegation is essentially different from default exclusion that is grounded in paternalism, as it respects children’s right to exercise autonomy in the sense of turning to their valued support resources (e.g. relationships) to aid them in making informed decisions.^14^

## Conclusion

Obtaining assent from children in paediatric oncology research is an ethically important practice that should be grounded in respect for the child’s emerging autonomy, integrity, and personhood – through active and meaningful participation. While concern for the child’s well-being is essential, it does not justify excluding them from the assent process. On the contrary, as an important moral value and a relational activity, children’s autonomy should be respected and actively supported, especially given the uncertainty of research and its non-therapeutic aim. Respecting children as persons means recognising them as individuals with their own perspectives and full human rights and prevents them from being treated as mere means to research ends. However, autonomy for children is not only a right. From the care ethics perspective we have applied in this article, not only autonomy as a right or well-being as a principle are relevant but also autonomy as a value and as a relational activity. To achieve this, researchers and research nurses need to develop skills that promote trust and avoid instrumentalisation of children, thereby strengthening children’s active and meaningful participation in decisions that concern them.
